# Downregulation of PPARγ by miR-548d-5p suppresses the adipogenic differentiation of human bone marrow mesenchymal stem cells and enhances their osteogenic potential

**DOI:** 10.1186/1479-5876-12-168

**Published:** 2014-06-14

**Authors:** Junkui Sun, Yisheng Wang, Yuebai Li, Guoqiang Zhao

**Affiliations:** 1 Department of Orthopaedic Surgery, the First Affiliated Hospital of Zhengzhou University, Zhengzhou, Henan 450052, China; 2 Basic Medical College, Zhengzhou University, Zhengzhou, Henan 450001, China

**Keywords:** miR-548d-5p, Peroxisome proliferator-activated receptor-γ, Bone marrow mesenchymal stem cells, Adipogenic differentiation

## Abstract

**Background:**

Human bone marrow mesenchymal stem cells (hBMSCs) are multipotent cells that can differentiate into a variety of cell types. Elevated expression of peroxisome proliferator-activated receptor-γ (PPARγ) promotes the adipogenic differentiation of hBMSCs, and reduces their osteogenic differentiation. MicroRNAs (miRNAs) have been shown to play important roles in the regulation of hBMSCs differentiation. Because bioinformatic analysis has indicated that PPARγ is a candidate target of miR-548d-5p, the aim of this study was to assess the impact of miR-548d-5p on the dexamethasone-induced adipogenic differentiation of hBMSCs.

**Methods:**

A quantitative RT-PCR (qRT-PCR) assay was used to compare miR-548d-5p expression levels in dexamethasone-induced hBMSCs and uninduced control cells. Oil red O staining, cellular triglyceride (TG) content, and the mRNA and protein levels of PPARγ and CCAAT/enhancer binding protein α (C/EBPα) were used to evaluate the adipogenic differentiation of hBMSCs. Alkaline phosphatase (ALP) activity and levels of osteocalcin (OCN) and Runx2 were used to evaluate the osteogenic potential of hBMSCs.

**Results:**

Compared with untreated cells, miR-548d-5p expression levels were downregulated during dexamethasone-induced adipogenic differentiation of hBMSCs. In contrast to the profuse Oil Red O staining in the cytoplasm of dexamethasone + scrambled miRNA-treated cells, there was limited staining in the cytoplasm of dexamethasone + miR-548d-5p-treated cells, indicating the absence of adipocytes. Moreover, compared with scrambled miRNA-treated cells, treatment with miR-548d-5p suppressed cellular levels of PPARγ and C/EBPα mRNA and protein, and cell TG content (*P* < 0.05). In contrast, compared with scrambled miRNA-treated cells, cellular levels of OCN and Runx2 mRNA and protein, as well as ALP activity, were significantly higher in miR-548d-5p-treated cells (*P* < 0.05). Western blot and luciferase reporter assays confirmed that miR-548d-5p directly targeted the 3′-untranslated region of PPARγ.

**Conclusions:**

miR-548d-5p is downregulated during dexamethasone-induced adipogenic differentiation of hBMSCs. By directly targeting and downregulating PPARγ, miR-548d-5p suppresses the dexamethasone-induced adipogenic differentiation of hBMSCs and enhances their osteogenic potential. Our findings suggest that miR-548d-5p has potential in the treatment of corticosteroid-induced osteonecrosis of the femoral head.

## Introduction

Human bone marrow mesenchymal stem cells (hBMSCs) are multipotent cells that can differentiate into a variety of cell types, including adipocytes, osteoblasts, and chondrocytes [1-3]. While hBMSCs differentiation is known to be modulated by a number of regulatory factors and complex signaling pathways [4,5], it remains largely uncharacterized. A variety of human diseases, such as osteoporosis, age-related bone loss, and osteonecrosis, have been linked to imbalances between adipogenic and osteogenic differentiation of hBMSCs [6,7]. For example, osteonecrosis of the femoral head (ONFH), resulting from increased intraosseous pressure and ischemia in the femoral head, is frequently observed in patients treated with elevated doses of corticosteroids [8].

Treatment of hBMSCs with pharmacological doses of glucocorticoids increases intracellular triglycerides and promotes adipogenic differentiation [9], a complex process involving a coordinated cascade of multiple transcription factors and signaling pathways. Induction of the adipogenic genes that ultimately give rise to the phenotype of terminally differentiated adipocytes is co-ordinated by CCAAT/enhancer binding protein α (C/EBPα) and peroxisome proliferator-activated receptor-γ (PPARγ) [10,11], a member of the ligand-activated nuclear receptor superfamily of transcription factors [10-12]. Elevated cellular levels of PPARγ promote the adipogenic differentiation of hBMSCs and inhibit their osteogenic differentiation, increasing cellular lipid levels and decreasing bone formation [13]. Consistent with this, down-regulation of PPARγ inhibits the adipogenic differentiation of rabbit BMSCs, and promotes their osteogenic differentiation potential, an effect that would potentially antagonize corticosteroid-induced ONFH [14,15].

MicroRNAs (miRNAs) are small non-coding RNAs that regulate gene expression by targeting complementary sequences located primarily within the 3′-untranslated regions (UTRs) of their target mRNAs [16-18]. miRNAs play well-characterized pivotal roles in a variety of biological processes, including cell fate determination in embryonic stem cells, cell proliferation, apoptosis, differentiation, morphogenesis, and carcinogenesis [19-22]. Additionally, miRNAs have been shown to participate in the regulation of adipogenic and osteogenic differentiation in hBMSCs [23-25].

Although bioinformatic analysis has implicated PPARγ as a candidate target of miR-548d-5p, the effect of miR-548d-5p on glucocorticoid-induced adipogenic differentiation of hBMSCs remains obscure. In the present study, we measured cellular levels of miR-548d-5p during dexamethasone-induced adipogenic differentiation of hBMSCs, and assessed the effect on this process of treatment of hBMSCs with miR-548d-5p.

## Materials and methods

### Cell culture

hBMSCs were purchased from Cyagen Biosciences Co. Ltd. (Guangzhou, China), cell identification by flow cytometry revealed that hBMSCs were positive for CD29, CD44 and CD105, negative for CD34 and CD45. They were cultured in low glucose complete DMEM culture medium (containing 10% fetal bovine serum, 100 kU/mL penicillin, 100 mg/L streptomycin, 50 mg/L vitamin C, 1 mmol/L L-glutamine, and 20 mmol/L HEPES) and incubated in a humidified atmosphere of 5% CO_2_ at 37°C. The third passage hBMSCs were used in this study.

### Dexamethasone-induced adipogenic differentiation

Dexamethasone was added to hBMSCs medium which requiring dexamethasone induction at a final concentration of 10^−7^ mol/L. The same concentration of dexamethasone was added to new medium each time it was replaced.

### miRNA transfection

The miR-548d-5p agomir (GMR-miR™ microRNA-548d-5p agomir) used in this study was synthesized by Shanghai GenePharma Co. Ltd. (Shanghai, China). Prior to transfection, hBMSCs were plated in six-well plates at a density of 1.5 × 10^5^ cells/well. Once cells reached ~50% confluence, transient transfections were conducted using Lipofectamine™2000 (Invitrogen, Carlsbad, CA, USA) following the manufacturer’s instructions, each well was transfected with 50nmol miR-548d-5p agomir.

### RNA extraction and quantitative RT-PCR

Total RNA was extracted from cells using a Total RNA Kit (OMEGA, Norcross, GA, USA), according to the manufacturer’s instructions. cDNA was synthesized using the RevertAid First Strand cDNA (Thermo Fisher Scientific, Waltham, MA, USA). qRT-PCR was carried out using SYBR Green I (TaKaRa, Dalian, China), and gene expression levels were normalized to GAPDH. All experiments were performed in triplicate. To verify mature miRNA expression levels, qRT-PCR was performed using a High-Specificity miR-548d-5p qRT-PCR Detection Kit (Stratagene Corp, La Jolla, CA, USA) in conjunction with an ABI 7500 thermal cycler, according to the manufacturer’s recommendations. The miR-548d-5p expression level was normalized to U6 small nuclear RNA (U6 snRNA), and measured using the comparative Ct (2^-ΔCt^) method. The primers for miR-548d-5p were 5’GTCGTATCCAGTGCAGGGTCCGAGGTATT CGCACTGGATACGACGGCAAAA3’ (RT primer), 5’TCCGAAAAAGTAATTGTGGT 3’ (forward), 5’GTGCAGGGTCCGAGGT 3’(reverse). The primers for U6 were 5’GTCGTATCCAGTGCAGGGTCCGAGGTATTCGCACTGGATACGACAAAATA3’ (RT primer), 5’TCCGATCGTGAAGCGTTC3’ (forward), 5’GTGCAGGGTCCGAGGT 3’ reverse).

### Oil red O staining

Oil red O staining was conducted at day 14 following dexamethasone treatment. Cells were washed twice with PBS and fixed with 10% formalin for 10 min at room temperature. After fixation, cells were stained with filtered oil red O solution for 1h at room temperature.

### Determination of cellular triglyceride (TG) content

Cellular TG content was determined using a Triglyceride Determination Kit (Applygen, Beijing, China) at day 14 of dexamethasone treatment. hBMSCs were collected, with a final cell density of 1 × 10^6^ cell/mL in each group. After centrifugation at 1000 r/min for 10 min, cells were washed twice with PBS and lysed with 1% TritonX-100 for 30 min. Then, 3 μL of cytochylema and 300 μL of working solution were added into 96-well plates, meanwhile set blank wells and calibration wells. Cells were incubated at 37°C for 5 min after blending, and the absorbance values were measured at a wavelength of 500 nm.

### ALP activity assay

ALP activity was determined using an enzyme-linked immunosorbent assay (ELISA) test kit (R&D Systems, Minneapolis, MN, USA) at day 6 of culture. hBMSCs were suspended in 1 mL buffer solution and centrifuged at 15 000 r/min for 15 min at 4°C after repeated freezing-thawing treatment to lyse the cells. Set standard wells on the ELISA plates and prepared standard solution. Forty microliters of diluent and 10 μL of supernatant were added into sample wells, and incubated at 37°C for 30 min after blending, meanwhile set blank wells. After washing 3 times, 50 μL of horseradish peroxidase was added into the sample wells, and incubated at 37°C for 30 min. After washing for three times, 100 μL of chromogenic solution was added and incubated at 37°C for 15 min. Finally, 50 μL of stop solution was added and absorbance values were measured at a wavelength of 450 nm.

### Dual-luciferase assay

By bioinformatic analysis using TargetScan (http://www.targetscan.org/) and miRBase (http://www.mirbase.org/), we suggest that PPARγ as a candidate target of miR-548d-5p. Human PPARγ 3′-UTR fragments containing putative binding sites for miR-548d-5p were amplified by PCR from human genomic DNA. Mutant PPARγ 3′-UTRs were obtained by overlap extension PCR. The fragments were cloned into a pmirGLO reporter vector (Promega), downstream of the luciferase gene, to generate the recombinant vectors pmirGLO-PPARγ-Wt and pmirGLO-PPARγ-Mut. For the luciferase reporter assay, hBMSCs were transiently co-transfected with miRNA (miR-548d-5p agomir or scrambled negative control) and reporter vectors (wild-type reporter vectors or mutant-type reporter vectors), using Lipofectamine™2000 (Invitrogen, Carlsbad, CA, USA). Luciferase activities were measured using a Dual-Luciferase assay kit (Promega, Madison, WI, USA) according to the manufacturer’s instructions at 48 h post-transfection.

### Western blotting

Total proteins of cultured cells were extracted using RIPA buffer containing phenylmethanesulfonylfluoride. Protein concentration was determined using the BCA protein assay kit (Beyotime, Haimen, China) according to the manufacturer’s protocol. Proteins were subjected to sodium dodecyl sulfate polyacrylamide gel electrophoresis and transferred onto polyvinylidene difluoride membranes. After blocking, the membranes were incubated overnight at 4°C with diluted (1:300) primary antibodies (monoclonal mouse anti-PPARγ or anti-C/EBPα or anti-OCN or anti-Runx2; Santa Cruz Biotechnology, Dallas, TEX, USA). Following extensive washing, the membranes were incubated with diluted (1:3000) horseradish peroxidase-conjugated rabbit anti-mouse IgG (Santa Cruz Biotechnology, Dallas, TEX, USA). Signals were determined using a chemiluminescence detection kit (Amersham Pharmacia Biotech, Piscataway, NJ, USA). An antibody against β-actin (Santa Cruz Biotechnology, Dallas, TEX, USA) served as endogenous reference.

### Statistical analysis

Data are presented as mean ± standard deviation. Data processing was performed by analysis of variance. Pairwise comparison among groups was performed using multiple comparisons tests. Statistical analysis was performed with SPSS 17.0 software (SPSS Inc., Chicago, IL, USA). Differences with *P* < 0.05 were considered statistically significant.

## Results

### Regulation of miR-548d-5p levels by dexamethasone during adipogenic differentiation of hBMSCs

**Figure 1:**
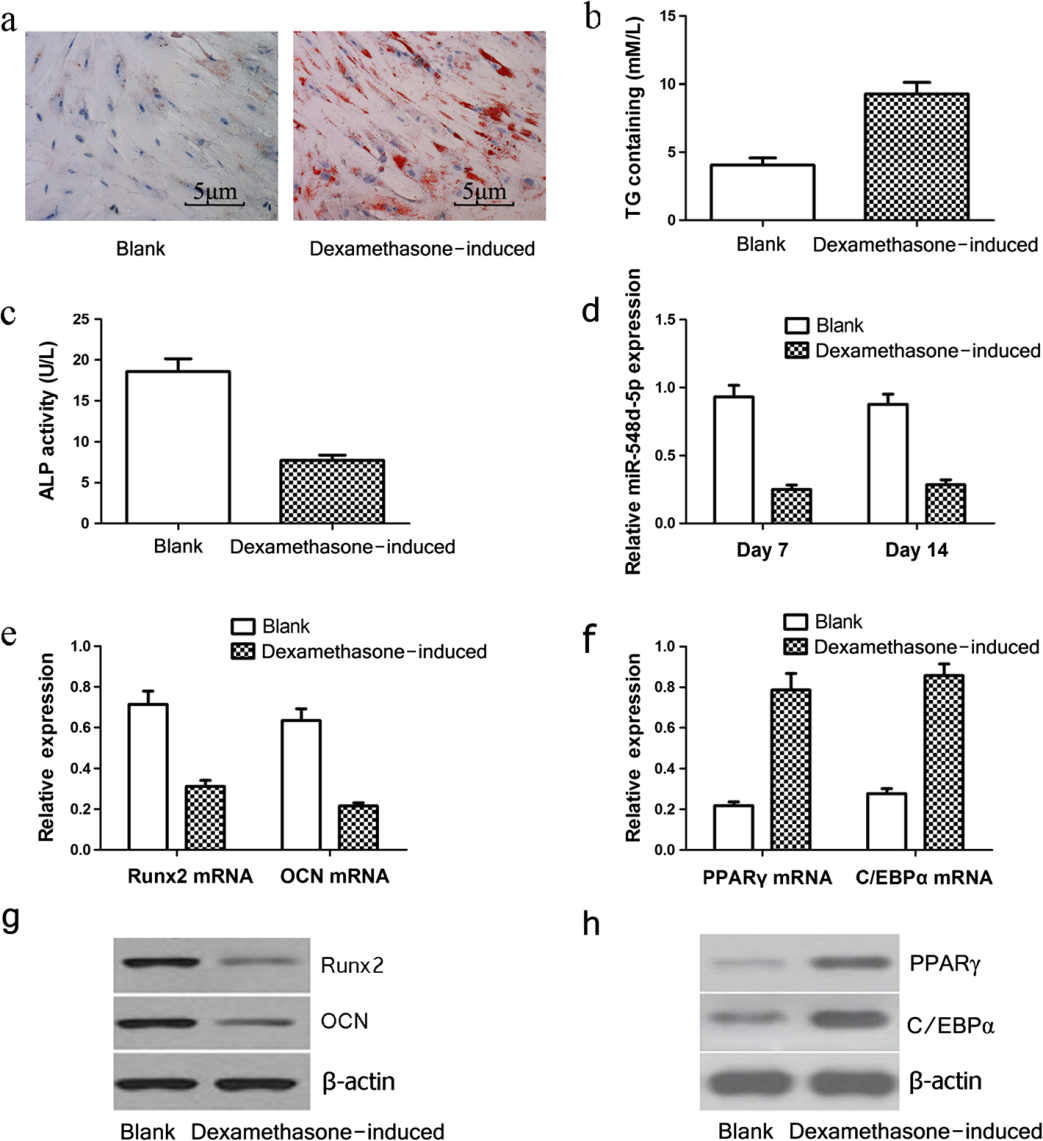
**Dexamethasone induces adipogenic differentiation of hBMSCs and downregulates miR-548d-5p expression.** Blank, non-induced hBMSCs; Dexamethasone-induced, hBMSCs induced with dexamethasone. **(a)** Oil red O staining showing more adipocytes were detectable in the cytoplasm of the cells in the dexamethasone-induced group than the Blank group at day 14. **(b)** TG content was measured at day 14. **(c)** ALP activity was measured using ELISA at day 6. **(d)** Expression levels of miR-548d-5p in the Dexamethasone-induced group and Blank group were measured by qRT-PCR at day 7 and day 14. U6 was used as an internal control. **(e,f)** qRT-PCR analysis of Runx2, OCN, PPARγ, and C/EBPα. The data were normalized to GAPDH. **(g,h)** Runx2, OCN, PPARγ, and C/EBPα protein levels were detected by Western blotting analysis, β-actin was used as a reference.

Based on studies showing that supraphysiological doses of glucocorticoids induce the differentiation of hBMSCs into adipocytes, 10^−7^ mol/L of dexamethasone was chosen as the effective dose [9]. Compared with non-induced (Blank) cells, hBMSCs cultured in the presence of dexamethasone had higher cytoplasmic oil red O staining (Figure 1a) and higher TG content (*P* < 0.05; Figure 1b) after 14 days, and decreased levels on day 6 of ALP, an early marker of osteogenic differentiation (*P* < 0.05; Figure 1c). Moreover, relative to cells in the Blank group, dexamethasone-treated hBMSCs had lower levels of miR-548d-5p (day 7 and day 14, *P* < 0.05; Figure 1d) and the osteogenic markers Runx2 and OCN (day 6, *P* < 0.05, Figure 1e, 1g). Furthermore, qRT-PCR (day 6, *P* < 0.05; Figure 1f) and western blotting (day 6, Figure 1h) showed that levels of the adipogenic transcription factors PPARγ and C/EBPα were higher in the dexamethasone-induced group than in the Blank group. Collectively, these results confirm the induction by dexamethasone of adipogenic differentiation in hBMSCs, and its inhibition of osteogenic differentiation in these cells. The induction of miR-548d-5p expression in dexamethasone-treated hBMSCs suggested to us that miR-548d-5p had a role in the regulation of their adipogenic differentiation.

### Overexpression of miR-548d-5p suppresses dexamethasone-induced adipogenic differentiation of hBMSCs

**Figure 2:**
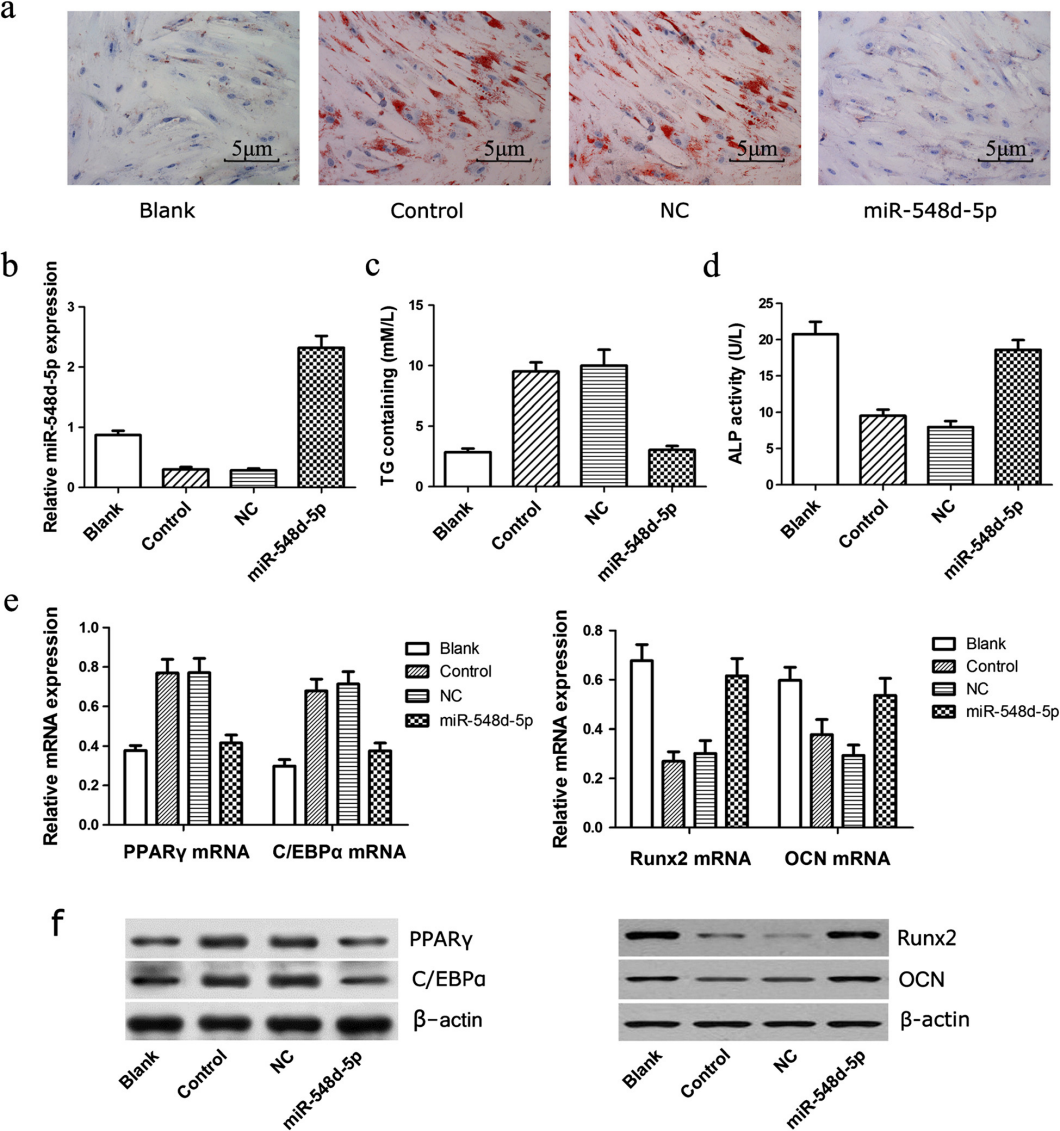
**Overexpression miR-548d-5p suppresses dexamethasone-induced adipogenic differentiation of hBMSCs.** Blank, non-transfected and non-induced cells; Control, non-transfected hBMSCs induced with dexamethasone; NC, cells transfected with scrambled miR-548d-5p negative control and induced with dexamethasone; miR-548d-5p, cells transfected with miR-548d-5p agomir and induced with dexamethasone. **(a)** Oil red O staining on day 14 of culture. **(b)** Mature miR-548d-5p expression levels after transfection, U6 was used as an internal control. **(c)** TG containing was measured at day 14. **(d)** The ALP activity in the Control and NC groups were significantly lower than in the Blank group (*P* < 0.05), but there were no significant differences between the miR-548d-5p and Blank groups (*P >* 0.05). **(e)** PPARγ, C/EBPα, Runx2, and OCN mRNA levels were detected by qRT-PCR analysis. The data were normalized to GAPDH. **(f)** PPARγ, C/EBPα, Runx2, and OCN protein levels were detected by western blotting analysis, β-actin was used as a reference.

We next sought to elucidate the role of miR-548d-5p during dexamethasone-induced adipogenic differentiation of hBMSCs by transfection studies using the miR-548d-5p agomir. Cells were divided into four groups, namely: Blank Group: untransfected, uninduced hBMSCs; Control Group: untransfected hBMSCs induced with dexamethasone; NC Group: scrambled agomir-transfected hBMSCs induced with dexamethasone; and miR-548d-5p Group: miR-548d-5p agomir-transfected hBMSCs induced with dexamethasone. qRT-PCR analysis showed that miR-548d-5p levels were eight-fold higher in the miR-548d-5p Group compared with the Control and NC groups (day 2, Figure 2b). Oil red O staining assay on day 14 of induction showed that, in contrast to cells in the Blank and miR-548d-5p groups, in which no or few cells stained positive, cells in the Control and NC groups contained reddish-orange fat droplets in their cytoplasm (Figure 2a). Consistent with this, TG content in the Blank and miR-548d-5p groups was significantly lower than in the NC and Control groups (day 14, *P* < 0.05; Figure 2c). Moreover, the higher ALP activity in the miR-548d-5p group relative to the NC and Control groups (day 6, *P* < 0.05; Figure 2d) suggested that miR-548d-5p maintained the osteogenic potential of hBMSCs. Compared with the NC and Control groups, overexpression of miR-548d-5p depleted PPARγ and C/EBPα at both the mRNA and protein levels (*P* < 0.05), and increased protein levels of the osteogenic markers Runx2 and OCN (*P* < 0.05) (day 6, Figure 2e, 2f). These results indicated to us that overexpression of miR-548d-5p suppressed the dexamethasone-induced adipogenic differentiation of hBMSCs and supported the osteogenic potential of these cells.

### PPARγ is direct target of miR-548d-5p

**Figure 3:**
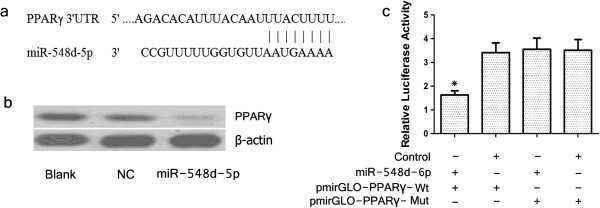
**PPARγ is a target of miR-548d-5p in hBMSCs. (a)** The putative miR-548d-5p seed region in the PPARγ 3’-UTR. **(b)** Western blotting analysis of PPARγ expression in transfected cells. β-actin was used as a reference. Blank, nontransfected cells; NC, cells transfected with scrambled miR-548d-5p negative control; miR-548d-5p, cells transfected with miR-548d-5p agomir. **(c)** Luciferase activity determined 48 h after transfection. Control, scrambled miRNA; miR-548d-5p, miR-548d-5p agomir; pmirGLO-PPARγ-Wt, wild-type pmirGLO-PPARγ; pmirGLO-PPARγ-Mut, mutant pmirGLO-PPARγ. *P < 0.05 compared with the control group.

Bioinformatic analysis using TargetScan and miRanda indicated that PPARγ is a candidate target of miR-548d-5p (Figure 3a), leading us to speculate as to whether miR-548d-5p could directly down-regulate PPARγ expression. Western blotting assay indicated that overexpression of miR-548d-5p substantially decreased cellular levels of PPARγ (Figure 3b). Consistent with this, we found that compared with cells in the NC group, luciferase activity was reduced by cotransfection of miR-548d-5p with the pmirGLO-PPARγ-Wt vector, but not the pmirGLO-PPARγ-Mut vector (Figure 3c). These results indicated that miR-548d-5p regulated PPARγ expression by directly targeting the 3′-UTR of PPARγ.

### Expression of PPARγ restores anti-dexamethasone-induced adipogenic differentiation function of miR-548d-5p

**Figure 4:**
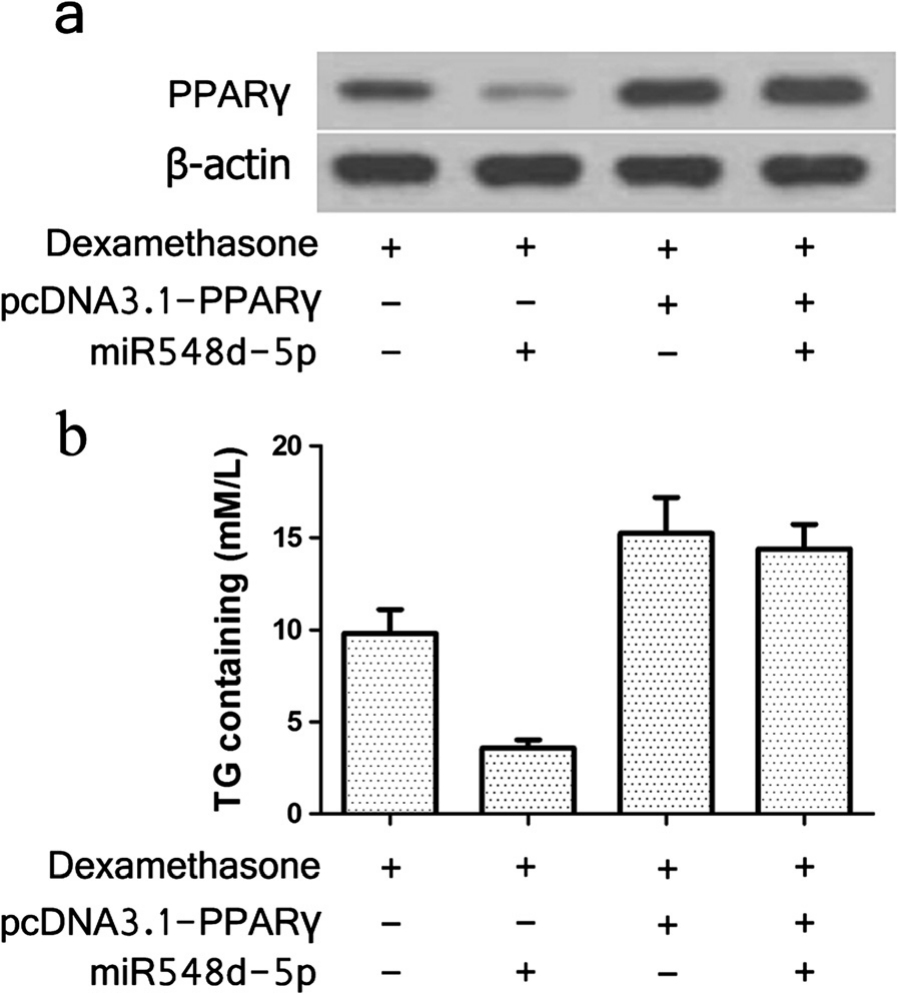
**Expression of PPARγ abrogates the anti-dexamethasone-induced adipogenic differentiation function of miR-548d-5p. (a)** Western blotting analysis of hBMSCs transfected with pcDNA3.1-PPARγ lacking the 3′-UTR sequence and/or miR-548d-5p. β-actin was used as a reference. **(b)** TG content was measured in hBMSCs transfected with pcDNA3.1-PPARγ lacking the 3′-UTR sequence and/or miR-548d-5p.

Western blotting analysis showed that cell levels of PPARγ were decreased in miR-548d-5p agomir-transfected hBMSCs, but not in cells co-transfected with miR-548d-5p agomir and PPARγ lacking the 3′-UTR sequence (pcDNA3.1-PPARγ) (Figure 4a). Moreover, we found that TG content was not decreased in hBMSCs co-transfected with pcDNA3.1-PPARγ and miR-548d-5p agomir (Figure 4b). These results indicated that PPARγ expression restores the anti-dexamethasone-induced adipogenic differentiation function of miR-548d-5p.

## Discussion

miRNAs play important roles in the regulation of hBMSCs differentiation. miR-141 and miR-200a, for example, have been reported to regulate osteoblast differentiation by targeting the BMP-2 signaling pathway [26]. Moreover, miR-346 positively regulates hBMSCs osteogenic differentiation by targeting GSK-3β and activating the Wnt/β-catenin pathway [27]. In the current study, we found that miR-548d-5p inhibits dexamethasone-induced adipogenesis in hBMSCs by targeting PPARγ. Our data indicate that miR-548d-5p overexpression down-regulated PPARγ levels by directly targeting the 3′-UTR of PPARγ mRNA. Luciferase reporter gene assays demonstrated that this effect was largely eliminated when bases in the PPARγ mRNA 3′-UTR targeted by miR-548d-5p were mutated.

PPARγ is one of the most important cell-fate-defining factors in hBMSCs. It has been shown to positively regulate adipogenesis [28,29], and is one of the earliest induced genes in preadipocytes [30]. Consistent with this, we found that PPARγ was induced at both the mRNA and protein levels during dexamethasone-induced adipogenesis in hBMSCs, an effect that was abrogated in the presence of transfected miR-548d-5p. These data indicated that miR-548d-5p overexpression suppressed PPARγ at both the mRNA and protein levels, and in doing so inhibited dexamethasone-induced adipogenic differentiation.

Runx2 is a master regulator of osteogenesis in hBMSCs, and was the first transcription factor to be shown to be required for determination of the osteoblast lineage [31]. Analogous to PPARγ in adipogenic differentiation, Runx2 mediates the effects of a variety of cytokines in determining the osteogenic differentiation of hBMSCs [32]. We found that expression of Runx2 and OCN were increased in the presence of miR-548d-5p, as was the activity of ALP, which plays an important role in the process of osteoblast differentiation of hBMSCs [33]. Collectively, these data implied that miR-548d-5p promotes the osteogenic potential of dexamethasone-induced hBMSCs.

In conclusions, miR-548d-5p is downregulated during dexamethasone-induced adipogenic differentiation of hBMSCs. miR-548d-5p inhibits steroid-induced adipogenesis of hBMSCs and maintains their osteogenic potential by targeting PPARγ. Our findings suggest that miR-548d-5p has potential in the treatment of corticosteroid-induced osteonecrosis of the femoral head.

## Abbreviations

miRNAs: MicroRNAs; hBMSCs: Human bone marrow mesenchymal stem cells; PPARγ: Peroxisome proliferator-activated receptor-γ; C/EBPα: CCAAT/enhancer binding protein α; TG: Triglyceride; ALP: Alkaline phosphatase; OCN: Osteocalcin; ONFH: Osteonecrosis of the femoral head; 3’-UTRs: 3’-untranslated regions; ELISA: Enzyme-linked immunosorbent assay.

## Competing interests

The authors have declared that no competing interest exists.

## Authors’ contributions

JS, YL and GZ performed and participated in analysis of laboratory experiments data. YW, YL and GZ participated in the design of experiments. YW, YL provided administrative support and funded experiments. JS, YW and GZ drafted the manuscript. All authors have contributed and approved the final manuscript.

## References

[B1] Chamberlain G Fox J Ashton B Middleton J Concise review: mesenchymal stem cells: their phenotype, differentiation capacity, immunological features and potential for homing Stem Cells 2007 25 2739 2749 10.1634/stemcells.2007-0197 17656645

[B2] Fink T Rasmussen JG Emmersen J Pilgaard L Fahlman A Brunberg S Josefsson J Arnemo JM Zachar V Swenson JE Frobert O Adipose-derived stem cells from the brown bear (Ursus arctos) spontaneously undergo chondrogenic and osteogenic differentiation in vitro Stem Cell Res 2011 7 1 89 95 10.1016/j.scr.2011.03.003 21497574

[B3] Phinney DG Prockop DJ Concise review: mesenchymal stem/multipotent stromal cells: the state of transdifferentiation and modes of tissue repair-current views Stem Cells 2007 25 11 2896 2902 10.1634/stemcells.2007-0637 17901396

[B4] Phimphilai M Zhao Z Boules H Roca H Franceschi RT BMP signaling is required for RUNX2-dependent induction of the osteoblast phenotype J Bone Miner Res 2006 21 637 646 10.1359/jbmr.060109 16598384 PMC2435171

[B5] Huang W Yang S Shao J Li YP Signaling and transcriptional regulation in osteoblast commitment and differentiation Front Biosci 2007 12 3068 3092 10.2741/2296 17485283 PMC3571113

[B6] Justesen J Stenderup K Ebbesen EN Mosekilde L T Steiniche T Kassem M Adipocyte tissue volume in bone marrow is increased with aging and in patients with osteoporosis Biogerontology 2001 2 165 171 10.1023/A:1011513223894 11708718

[B7] Nuttall ME Gimble JM Is there a therapeutic opportunity to either prevent or treat osteopenic disorders by inhibiting marrow adipogenesis Bone 2000 27 2 177 184 10.1016/S8756-3282(00)00317-3 10913909

[B8] Tan G Kang PD Pei FX Glucocorticoids affect the metabolism of bone marrow stromal cells and lead to osteonecrosis of the femoral head: a review Chin Med J (Engl) 2012 125 134 139 22340480

[B9] Yin L Li YB Wang YS Dexamethasone-induced adipogenesis in primary marrow stromal cell cultures: mechanism of steroid-induced osteonecrosis Chin Med J (Engl) 2006 119 581 588 16620700

[B10] Li H Li T Wang S Wei J Fan J Li J Han Q Liao L Shao C Zhao RC miR-17-5p and miR-106a are involved in the balance between osteogenic and adipogenic differentiation of adipose-derived mesenchymal stem cells Stem Cell Res 2013 10 313 324 10.1016/j.scr.2012.11.007 23399447

[B11] Wu Z Xie Y Bucher NR Farmer SR Conditional ectopic expression of C/EBP beta in NIH-3T3 cells induces PPAR gamma and stimulates adipogenesis Genes Dev 1995 9 2350 2363 10.1101/gad.9.19.2350 7557387

[B12] Rosen ED Walkey CJ Puigserver P Spiegelman BM Transcriptional regulation of adipogenesis Genes Dev 2000 14 1293 1307 10837022

[B13] Lowell BB PPARgamma: an essential regulator of adipogenesis and modulator of fat cell function Cell 1999 99 3 239 242 10.1016/S0092-8674(00)81654-2 10555139

[B14] Wang YS Li JF Liu M Zhao GQ Hao LY Li YB Inhibition of peroxisome proliferator-activated receptor-γ in steroid-induced adipogenic differentiation of the bone marrow mesenchymal stem cells of rabbit using small interference RNA Chin Med J (Engl) 2014 127 1 130 136 24384438

[B15] Liu M Wang YS Li YB Zhao GQ Construction and identification of the recombinant adenovirus vector carrying a small interfering RNA targeting the peroxisome proliferator-activated receptor-γ Chin Med J (Engl) 2012 125 671 675 22490494

[B16] Karp X Ambros V Developmental biology Encouraging miRNAs in cell fate signaling Science 2005 310 1288 1289 10.1126/science.1121566 16311325

[B17] He H Jazdzewski K Li W Liyanarachchi S Nagy R Volinia S Calin GA Liu CG Franssila K Suster S Kloos RT Croce CM de la Chapelle A The role of microRNA genes in papillary thyroid carcinoma Proc Natl Acad Sci 2005 02 52 19075 19080 16365291 10.1073/pnas.0509603102 PMC1323209

[B18] Ambros V The functions of animal microRNAs Nature 2004 431 350 355 10.1038/nature02871 15372042

[B19] Kim VN Nam JW Genomics of microRNA Trends Genet 2006 22 3 165 173 10.1016/j.tig.2006.01.003 16446010

[B20] Kwak PB Iwasaki S Tomari Y The microRNA pathway and cancer Cancer Sci 2010 101 11 2309 2315 10.1111/j.1349-7006.2010.01683.x 20726859 PMC11159795

[B21] Bartel DP MicroRNAs: genomics, biogenesis, mechanism, and function Cell 2004 116 2 281 297 10.1016/S0092-8674(04)00045-5 14744438

[B22] Farazi TA Spitzer JI Morozov P Tuschl T miRNAs in human cancer J Pathol 2011 223 2 102 115 10.1002/path.2806 21125669 PMC3069496

[B23] Laine SK Alm JJ Virtanen SP Aro HT Laitala-Leinonen TK MicroRNAs miR-96, miR-124, and miR-199a regulate gene expression in human bone marrow-derived mesenchymal stem cells J Cell Biochem 2012 113 2687 2695 10.1002/jcb.24144 22441842

[B24] Eskildsen T Taipaleenmaki H Stenvang J Abdallah BM Ditzel N Nossent AY Bak M Kauppinen S Kassem M MicroRNA-138 regulates osteogenic differentiation of human stromal (mesenchymal) stem cells in vivo Proc Natl Acad Sci U S A 2011 08 6139 6144 21444814 10.1073/pnas.1016758108 PMC3076836

[B25] Huang J Zhao L Xing L Chen D MicroRNA-204 regulates Runx2 protein expression and mesenchymal progenitor cell differentiation Stem Cells 2010 28 357 364 20039258 10.1002/stem.288 PMC2837600

[B26] Itoh T Nozawa Y Akao Y MiRNA-141 and -200a are involved in bone morphogenetic protein-2-induced mouse pre-osteoblast differentiation by targeting distal-less homeobox 5 J Biol Chem 2009 284 29 19272 19279 10.1074/jbc.M109.014001 19454767 PMC2740552

[B27] Wang Q Cai J Cai XH Chen L miR-346 Regulates Osteogenic Differentiation of Human Bone Marrow-Derived Mesenchymal Stem Cells by Targeting the Wnt/b-Catenin Pathway PLoS One 2013 8 9 e72266 10.1371/journal.pone.0072266 24023731 PMC3762871

[B28] Muruganandan S Roman AA Sinal CJ Adipocyte differentiation of bone marrow-derived mesenchymal stem cells: cross talkwith the osteoblastogenic program Cell Mol Life Sci 2009 66 2 236 253 10.1007/s00018-008-8429-z 18854943 PMC11131547

[B29] Takada I Kouzmenko AP Kato S Wnt and PPARγ signaling in osteoblastogenesis and adipogenesis Nat Rev Rheumatol 2009 5 442 447 10.1038/nrrheum.2009.137 19581903

[B30] Wu Z Rosen ED Brun R Hauser S Adelmant G Troy AE McKeon C Darlington GJ Spiegelman BM Cross-regulation of C/EBP alpha and PPAR gamma controls the transcriptional pathway of adipogenesis and insulin sensitivity Mol Cell 1999 3 2 151 158 10.1016/S1097-2765(00)80306-8 10078198

[B31] Komori T Regulation of osteoblast differentiation by Runx2 Adv Exp Med Biol 2010 658 43 49 19950014 10.1007/978-1-4419-1050-9_5

[B32] Jeon MJ Kim JA Kwon SH Kim SW Park KS Activation of peroxisome proliferator activated receptor- gamma inhibits the Runx2- mediated transcription of osteocalcin in osteoblasts J Biol Chem 2003 278 23270 23277 10.1074/jbc.M211610200 12704187

[B33] Salasznyk RM Klees RF Boskey A Plopper GE Activation of FAK is necessary for the osteogenic differentiation of human mesenchymal stem cells on laminin-5 J Cell Biochem 2007 100 2 499 514 10.1002/jcb.21074 16927379

